# Current Photodynamic Therapy for Glioma Treatment: An Update

**DOI:** 10.3390/biomedicines12020375

**Published:** 2024-02-06

**Authors:** David Aebisher, Agnieszka Przygórzewska, Angelika Myśliwiec, Klaudia Dynarowicz, Magdalena Krupka-Olek, Andrzej Bożek, Aleksandra Kawczyk-Krupka, Dorota Bartusik-Aebisher

**Affiliations:** 1 Department of Photomedicine and Physical Chemistry, Medical College of the Rzeszów University, 35-959 Rzeszów, Poland; 2 English Division Science Club, Medical College of the Rzeszów University, 35-025 Rzeszów, Poland; ap117623@stud.ur.edu.pl; 3 Center for Innovative Research in Medical and Natural Sciences, Medical College of the Rzeszów University, 35-310 Rzeszów, Poland; amysliwiec@ur.edu.pl (A.M.); kdynarowicz@ur.edu.pl (K.D.); 4 Clinical Department of Internal Medicine, Dermatology and Allergology, Medical University of Silesia in Katowice, M. Sklodowskiej-Curie 10, 41-800 Zabrze, Poland; magda.krupka94@gmail.com (M.K.-O.); andrzej.bozek@sum.edu.pl (A.B.); 5 Department of Internal Medicine, Angiology and Physical Medicine, Center for Laser Diagnostics and Therapy, Medical University of Silesia in Katowice, Batorego 15 Street, 41-902 Bytom, Poland; 6 Department of Biochemistry and General Chemistry, Medical College of the Rzeszów University, 35-025 Rzeszów, Poland; dbartusikaebisher@ur.edu.pl

**Keywords:** glioma, PDT, cancer, treatment

## Abstract

Research on the development of photodynamic therapy for the treatment of brain tumors has shown promise in the treatment of this highly aggressive form of brain cancer. Analysis of both in vivo studies and clinical studies shows that photodynamic therapy can provide significant benefits, such as an improved median rate of survival. The use of photodynamic therapy is characterized by relatively few side effects, which is a significant advantage compared to conventional treatment methods such as often-used brain tumor surgery, advanced radiotherapy, and classic chemotherapy. Continued research in this area could bring significant advances, influencing future standards of treatment for this difficult and deadly disease.

## 1. Introduction

Gliomas are internal brain tumors originating from neuroglial progenitor cells [1,2]. They are highly heterogeneous tumors that are resistant to treatment [3,4]. Gliomas account for approximately 80% of the malignant intracranial tumors diagnosed [5,6,7,8]. Although gliomas are relatively rare in the population, they are associated with significant mortality with a 5-year relative survival rate of approximately 5% [9,10,11,12]. Gliomas invade and attack brain parenchyma, white matter tracts, and perivascular spaces [13]. The glioma tumor microenvironment (TME) consists of stromal cells, tumor components, and innate and adaptive immune cells, and these cells, along with the extracellular matrix, regulate and communicate intracellularly to promote TME formation. The immune microenvironment plays an important role in the development of glioma [14]. Patients with glioma are usually diagnosed at the symptomatic stage of the disease [15]. Although there is currently no cure for malignant glioma, researchers around the world continue to pursue a deeper understanding of the factors that influence glioma development and response to treatment [16]. Despite advances in neurosurgical technology and techniques, the survival rate of glioma patients has remained relatively unchanged in recent years, and thus improving the efficacy of glioma treatment is an urgent task in medicine. The main treatments for gliomas include surgery, radiotherapy, and chemotherapy [17,18,19,20,21]. Unfortunately, these methods are often associated with difficulties resulting from incomplete tumor resection and recurrence [22,23,24]. In such situations, photodynamic therapy (PDT) is becoming increasingly popular as an advanced therapeutic strategy, characterized by fewer side effects, minimal toxicity, and more controlled treatment [24,25,26]. The first studies on the use of PDT in high-grade gliomas showed promising results, such as increasing the median survival and extending the 2-year survival of patients from 18% to 28% [27,28,29,30,31]. In the initial phase of the development of PDT in oncology, the main goal was the complete removal of localized tumors, but over time, the clinical application of PDT in cancer treatment began to change [32,33,34,35,36]. Photodynamic therapy can be combined with additional anticancer therapies used in the clinic [37,38,39,40,41,42,43]. Successful treatment of glioma is hampered by many obstacles, including the immunosuppressive tumor microenvironment, the blood–brain barrier, and high heterogeneity [44]. Therefore, overcoming sufficient drug delivery and drug targeting to the glioma area is the key to obtaining an efficient treatment [45]. Continuing research on this innovative approach in the fight against malignant gliomas is certainly a step in the right direction and may result in further important discoveries. Novelty in our review refers to the unique perspective regarding the application of PDT in clinics. Photodynamic therapy addresses some hindrances in glioma treatment, e.g., the heterogeneous nature of treatment sites, and is in need of improvements in light delivery, imaging methods for determining PDT efficiency, and treatment planning.

## 2. Methodology

Publications written in English on the topic of PDT for glioma were searched for in PubMed and Research Gate. The keywords used for the search were: glioma; PDT; cancer; and treatment. Our article selection was based on the following criteria: (1) recent major achievements in this field; (2) current debates; and (3) ideas for future development of PDT for glioma. The papers not selected for this review included (1) technical reports, (2) papers not written in English, (3) clinical cases, and (4) PDT performed in vitro.

## 3. Standard Strategies for the Treatment of Gliomas

The greatest successes in the treatment of brain tumors are achieved by centers that implement multifaceted treatments of these types of tumors with participation of specialists in neurosurgery, radiology, pathology, radiation oncology, and neuro-oncology. There are three established guidelines for glioma treatment: surgery, radiotherapy, chemotherapy, and targeted therapy [46,47].

### 3.1. Surgery

The use of surgery in eradicating glioma faces problems of complete resection of glioma. These current problems are associated with imaging techniques to differentiate glioma and healthy brain tissue, resection extent, neurological function preservation, and tumor margin evaluation [48,49].

Despite inevitable recurrences, tumor resection is still an important element in prolonging patients’ lives. Technological advances that enable real-time visualization and quantification of tumorous and non-tumorous tissue are critical for brain tumor surgery [50]. Therefore, surgical planning by carefully observing the relationship between the area of glioma invasion and the eloquent area of connecting fibers is crucial. Neurosurgeons typically detect the eloquent area using functional MRI and identify the connecting fiber using diffusion tensor imaging [51]. Studies suggest that more extensive surgical resection may be associated with improved life expectancy in both low-grade and high-grade glioma patients [52,53]. Treatment strategies for patients with intracerebral glioma must be patient-specific. Studies indicate that maximal resection is desirable in patients with low-grade gliomas while preserving neurologic function. In patients with high-grade gliomas, the benefits of aggressive resection are more difficult to confirm. Patients who are relatively young and have tumors of significant mass are those who benefit most from aggressive resection [54]. In the recent neurosurgical literature, neurological outcomes were most frequently reported, followed by activities of daily living, seizure outcomes, neurocognitive outcomes, and Health-Related Quality of Life (HRQoL) outcomes following glioma surgery [55].

### 3.2. Radiotherapy

The use of conventional radiotherapy (RT) is the mainstay for the treatment of glioma. Although this methodology is advanced, it is also faced with problems. Despite the use of radiotherapy, chemotherapy, and surgical resection, glioma retains a poor prognostic value and no significant improvement in survival rates has been observed over the last 20 years [56].

Fractionated local irradiation is the main way to treat glioma. A standard radiation dose of 60 Gy is administered in more than 30 fractions of 1.8–2 Gy. The therapy is targeted at the diseased area with a 1–3 cm margin to effectively treat the infiltrating tumor [52]. Clinical RT was designed to target the majority of remaining glioma cells in healthy brain tissue with fewer side effects [57]. It is believed that the post-irradiation microenvironment is not suitable for the survival of cancer cells (tumor bed effect). However, resistance to radiotherapy remains a serious problem [58]. New techniques for delivering radiotherapy to the tumor are currently under investigation [59].

### 3.3. Chemotherapy

Chemotherapy is used before surgery in order to shrink the tumor prior to or after surgery to destroy the remaining cancer cells. To destroy glioma, high doses of chemo drugs are administered and, as a consequence, many healthy cells are also destroyed; stem cells can be transplanted into the patient [60].

Currently, temozolomide and chloroethyl nitroureas are mainly used to treat glioma with chemotherapy. Unfortunately, drugs administered systemically do not usually achieve high concentrations in the central nervous system and tumor area, leading to significant systemic side effects. Side effects of chemotherapy on the central nervous system include acute encephalopathy, leukoencephalopathy, cerebellar dysfunction, and spinal cord toxicity. Additionally, side effects may also occur in the peripheral nervous system, manifesting as chemotherapy-induced peripheral neuropathic pain [61,62]. Temozolomide (TMZ) is a common alkylating chemotherapeutic agent used to treat brain tumors such as glioma and anaplastic astrocytoma [63]. Temozolomide has a small molecular mass and this alkylating derivative directly damages brain tumor cells by methylating DNA [64]. The key cytotoxic effect is the formation of O^6^-methylguanine, which causes apoptosis, autophagy, and cell senescence [65,66]. Although TMZ therapy has shown gradual improvement in the treatment of high-grade gliomas, for most patients it is mainly a palliative treatment. In fact, in newly diagnosed glioma patients, the median survival benefit with TMZ plus radiation therapy is only 2.5 months compared with radiation therapy alone. Recent studies also suggest that 60–75% of glioma patients derive no benefit from TMZ treatment [67]. Most patients do not respond to TMZ during treatment. Activation of DNA repair pathways is the main mechanism of this phenomenon, which disconnects TMZ-induced O^6^-methylguanine adducts and restores genome integrity. Current knowledge in the field of oncology adds several other new resistance mechanisms, such as the involvement of miRNAs, drug efflux transporters, gap junction activity, the emergence of glioma stem cells, and the enhancement of cell survival autophagy [68]. Chloroethyl nitroureas (CNU) are DNA alkylating agents. In clinical practice, the most commonly used CNUs are lomustine, carmustine, and fotemustine [69].

In patients with WHO grade 4 gliomas and astrocytomas who have relapsed, lomustine chemotherapy is the standard treatment [70]. Lomustine, also known as CCNU, is an alkylating agent from the nitrosourea family [71,72]. The most common side effects are thrombocytopenia, with neutropenia and lymphocytopenia occurring less frequently [73,74,75]. Carmustine is used both in the initial diagnosis of glioma and in the case of tumor recurrence, both by intravenous administration [76]. The use in clinical practice is lower due to particularly long-lasting bone marrow suppression and persistent lung toxicity [77,78]. The developed biodegradable carmustine wafer may prove significant, enabling the administration of high doses of drugs [79,80]. Fotemustine has been used in the treatment of melanoma and is currently being tested for its effectiveness in the treatment of recurrent malignancy glioma [81].

### 3.4. Targeted Treatment

Tumor targeting as a novel strategy for glioma and targeted therapy uses drugs or other substances to identify and destroy cancer cells without damaging normal healthy cell survival and overall survival [82].

Pathways involved in tumor growth and processes of invasion and angiogenesis represent the main processes in glioma. The goal is to individualize treatment based on the specific molecular abnormalities of a given tumor [52]. Most drugs that target growth and survival pathways as monotherapies have failed to demonstrate survival benefits in populations of glioma patients. Targeting multiple signaling pathways with multi-target kinase inhibitors or combinations of single-target kinase inhibitors may increase treatment efficacy [83]. Main targets, including p53, retinoblastoma, and epidermal growth factor receptor gene, have been demonstrated to be ineffective. Research continues in immunotherapy-based treatments using immune checkpoint molecules, macrophages, and dendritic cells within the tumor microenvironment [84]. In recent years, CTLA-4 inhibitors have shown good effects in cancer immunotherapy. Tremelimumab is a monoclonal antibody against CTLA4 that has demonstrated positive responses in many published clinical trials when combined with the PD-1/PD-L1 blockade. However, in glioma, several studies have shown that antibodies anti-CTLA-4 and PD-1 show no survival benefit when compared to standard chemotherapy [85,86,87,88]. Furthermore, no obvious benefit was obtained with neoadjuvant nivolumab in resectable glioma [89]. Similarly, a phase III trial comparing nivolumab (anti-PD-1 blocking antibody) with bevacizumab (anti-VEGF blocking antibody) in patients with recurrent glioma showed no benefit from nivolumab and resulted in a similar median overall survival (mOS 9.8 vs. 10.0 months) [90,91]. Nevertheless, the paucity of effective antigenic targets remains a significant obstacle to the safe and effective treatment of gliomas with relatively low mutational load with immunotherapy [92]. Merely reducing the size of the tumor is not sufficient because recurrence and rapid progression of the tumor will ultimately kill the patient. There is a trend to incorporate immunotherapy into multimodal treatments, including radiotherapy and chemotherapy in patients with glioma because the effects of individual treatments may enhance each other [93]. Bevacizumab treatment is administered intravenously and this treatment with bevacizumab appears to not significantly improve overall survival in patients with newly diagnosed glioma [66,94]. The use of bevacizumab with irinotecan is also being investigated [95]. The most common side effects of bevacizumab include hypertension and leukopenia [96,97]. There is no evidence of differences in the effectiveness of chemotherapy depending on the age, sex, histology, functional status, or extent of resection [98].

## 4. Photodynamic Therapy

### 4.1. Mechanism of Tumor Destruction Using the PDT Method

**Figure 1:**
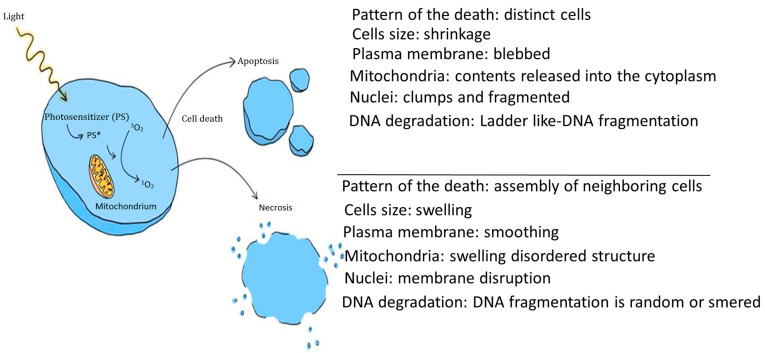
Mechanism of cell death after photodynamic therapy as a result of apoptosis and necrosis.

Photodynamic therapy employs photosensitizer molecules (PSs) that are selectively introduced into cancer cells. There are two key mechanisms of action in PDT. In the first mechanism (Type I), a photon absorbed by a photosensitizer molecule causes its excitation from the singlet ground state to the singlet excited state (PS*). The photosensitizer then inter-system crosses to the excited triplet state where it can undergo electron transfer reactions with oxygen or biomolecules in the environment. In the second mechanism (Type II), the photosensitizer in the excited triplet state transfers energy directly to the oxygen molecule to produce singlet oxygen (^1^O_2_). This ROS is characterized by a short lifetime and diffusion distance. Singlet oxygen is an electrophile and reacts rapidly with double bonds and heteroatoms such as sulfur. These reactions oxidatively damage the membranes of intracellular organelles such as mitochondria, lysosomes, and the endoplasmic reticulum [99,100,101]. This can promote irreversible destruction of cancer tissue primarily by apoptosis and/or necrosis (Figure 1). Another mechanism involves anti-vascular effects with the activation of PSs, provoking destruction of tumor vasculature. The third mechanism involves the activation of the immune response against cancer cells through acute inflammatory processes and the release of cytokines into the tumor. The advantage of one pathway over the other depends on the parameters used in therapy and the patient’s health condition [102,103,104,105,106,107,108].

### 4.2. PDT in Cancer Treatment

Clinical applications of PDT in dermatology, ophthalmology, urology, gastroenterology, gynecology, neurosurgery, and pneumology have shown encouraging results in the treatment of human cancers [103]. It is less invasive, target-specific, and has reduced cytotoxicity towards normal cells and tissues, which translates into fewer side effects [109,110,111]. Photodynamic therapy is selective as only diseased tissues that have accumulated PSs are irradiated. The idea of using PDT as a new treatment strategy appeared at the beginning of the 20th century [112,113,114]. Photodynamic therapy is a two-step procedure. First, a drug that absorbs light is administered to the patient. Then, after a period of time called the drug-light interval, the target tissue is irradiated. The drug is inactive in the dark, but upon electronic excitation, it transfers energy to molecular oxygen in the Type II process described previously [109,115,116,117,118,119,120]. The molecular structure and chemical properties of a PS determine the required wavelength of light, effective doses, and mechanism of action. The selection of an appropriate PS is one of the most important factors in achieving the intended effect [103,121]. Although the results of PDT vary depending on the type of cancer, its properties make it efficient in therapies [122].

### 4.3. Photodynamic Therapy in the Treatment of Brain Gliomas

Photodynamic therapy is applied for the treatment of neurological diseases and many types of brain tumors [123]. Photodynamic therapy has fewer side effects compared to chemotherapy and radiotherapy on the brain. After administration, the concentration of PSs is higher in glioma cells than in healthy tissue [31,124]. When combined with surgical resection, PDT for the treatment of gliomas, or alternatively as a stand-alone treatment strategy, has had some success in extending median patient survival compared to surgery alone [125]. The immunological effects of PDT are of particular interest given recent studies demonstrating the importance of these processes in glioma [126,127]. However, the use of this method in the treatment of gliomas also has some limitations. The main drawback is the development of resistance to PDT in tumors. Several mechanisms are known to be involved in the development of cellular defense against the cytotoxic effects of PDT, including activation of antioxidant enzymes, drug efflux pumps, PS degradation, and overexpression of chaperones [128].

DNA repair may aid in glioma resistance to PDT; however, this has not been further explored to date [129]. Many biological barriers may have an influence on the results of glioma PDT [9]. These include technical limitations of light delivery [130]. However, the problem of delivering light to the tumor, at least in some cases, can be solved by using implantable devices that enable light delivery during PDT, or near-infrared lasers that allow tissue penetration of up to 3 cm [9]. The effectiveness of glioma-PDT is based on the activation of PSs accumulated in the tumor with light. However, insufficient accumulation of PSs in the tumor severely limits the success of PDT [131]. The blood–brain barrier (BBB) is a significant limitation of PS transport to the area of postoperative resection, where brain tumor recurrence most often occurs [132,133]. In order to develop the “ideal photosensitizer”, there is still a need for new photodynamic agents with improved photophysical and photobiological properties [134]. Recent research has also led to the discovery of profound genetic heterogeneity among glioma cells that includes the adaptation to ROS. Therefore, tumor heterogeneity and the associated difference in sensitivity to ROS-producing therapeutic agents must be taken into account when designing PDT protocols to predict outcomes [135]. Moreover, there are no standard guidelines for PDT treatment protocols, and it is known that the selection of parameters affects the quality of treatment. Further observations are needed to further assess how PDT will reduce morbidity and mortality [136,137,138].

### 4.4. Nanoparticles for Glioma and PDT

To improve the results of brain tumor treatment, the use of nanoparticles containing a photosensitizer may be a promising strategy [9,139,140]. They are characterized by low cytotoxicity and excellent light absorption, which make them excellent agents for improving multifunctionality for imaging and treatment [141,142]. Numerous studies are currently underway to discover a nanoparticle with optimal properties for the treatment of glioma. Comincini et al. developed nanoparticles equipped with the photosensitizer berberine and showed that PDT using this system was responsible for an increase in early and late apoptosis of cancer cells without detecting any cytotoxic effect on healthy tissue [143]. Liu et al. constructed glycolipid-like micelles containing indocyanine green and demonstrated that it has the ability to improve drug delivery to neovascular endothelial cells and tumor cells and increase the effectiveness of phototherapy [144]. Teng et al. created nanoparticles by combining Indocyanine-Green with Chlorin-e6 on the surface of superparamagnetic iron oxide nanoparticles (SPIONs) and showed that they significantly increased local control of recurrent cancer and that they could be detected by NIR imaging, creating the potential for their use also in surgical oncology [145].

Research on upconversion nanoparticles (UCN) with covalently attached PSs has been conducted [146,147,148,149,150,151,152,153,154,155,156,157]. Upconversion nanoparticles are nanotransducers (NT) that absorb near-infrared light (NIR) and emit visible light (fluorescence) at wavelengths absorbed by PSs.

### 4.5. Photosensitizers Used in PDT

Obtaining new photosensitizers is a promising direction of research on photodynamic therapy (PDT) used in the diagnosis and treatment of cancer. However, for a photosensitizer to be effectively used in PDT, it must meet many important criteria. Here are the key conditions that such a photosensitizer should meet:

1. Selective accumulation in cancer tissue: One of the main factors influencing the effectiveness of PDT is the ability of the photosensitizer to selectively accumulate in cancer cells. An effective photosensitizer should preferentially accumulate in tumor tissue, minimizing absorption in healthy structures.
2. Availability and chemical properties: It is important that the photosensitizer is readily available as a pure compound and its exact chemical properties, such as reactivity and stability, should be thoroughly tested and documented. This ensures control over the synthesis process and guarantees safety and effectiveness of use.
3. No phototoxic effect in healthy tissue: It is extremely important that the photosensitizer does not cause a phototoxic effect in healthy tissue. This means that its effect should only be controlled and activated in the presence of light in the affected area.
4. High absorption coefficient in the range of 600–800 nm: the effectiveness of PDT is related to the absorption of light by the photosensitizer in the appropriate spectral range, most often in the range of 600–800 nm.
5. No overlap of absorption bands with other tissue components: The absorption band of the photosensitizer should not overlap the absorption bands of endogenous pigments such as melanin or hemoglobin, or the absorption bands of water in the near-infrared region. This avoids disruptions in the action of the photosensitizer.
6. Efficient production of ^1^O_2_ or radicals: the photosensitizer should have a high quantum yield of ^1^O_2_.
7. Optimal pharmacokinetic properties: the photosensitizer and its photoproducts should have appropriate pharmacokinetic properties, such as degradation, excretion, and bioavailability to ensure the effectiveness of the therapy.
8. Low side effects and easy elimination from the body: the therapeutic value of a photosensitizer is greater if it does not cause significant side effects and is easily eliminated from the body to avoid long-term toxic effects after PDT therapy.

**Figure 2:**
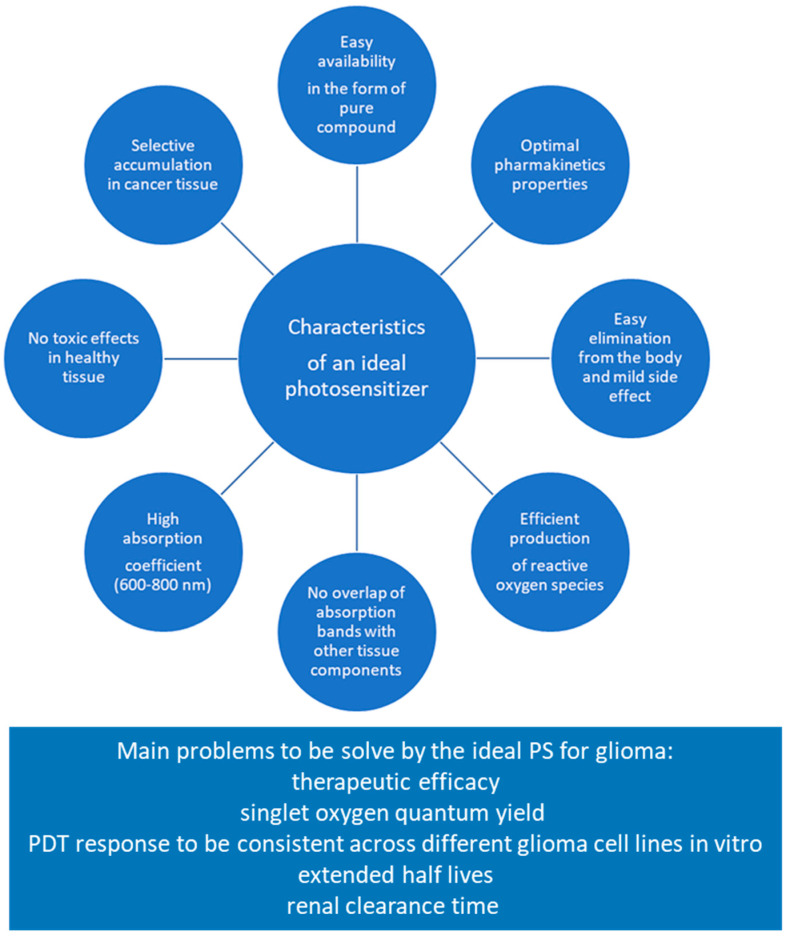
Characteristics of an ideal photosensitizer.

Figure 2 shows characteristics of an ideal photosensitizer. 

Meeting these criteria is crucial for the effectiveness and safety of using photosensitizers in photodynamic therapy, especially in the context of cancer treatment [158,159,160,161,162,163]. The historical development of photosensitizers in three subsequent generations is important in the context of PDT, bringing hope for further increasing its effectiveness and minimizing possible side effects.

First-generation PSs demonstrated effective PDT in the treatment of glioma [164,165] as well as limitations in the treatment of glioma resulting from their structure. First-generation PSs are limited not only by their low therapeutic effectiveness and low quantum yield of singlet oxygen [165]; first-generation PSs exhibited several limiting features that hampered their usefulness in the treatment of glioma. Therefore, it was justified to develop second-generation photosensitizers to increase the effectiveness of photodynamic therapy for glioma. Photosensitizers of the second generation have more efficient ROS production and enhanced tumor selectivity with limited adverse effects [166].

The first generation of photosensitizers includes porfimer sodium and hematoporphyrin-pioneered PDT. Although they are effective, they have limitations related to selectivity, and side effects in healthy tissues have been significant. In response to these limitations, second-generation photosensitizers were developed, such as derivatives of chlorin, bacteriochlorins, and phthalocyanines. They are characterized by stronger absorption in the deep red region of the spectrum, which allows for activation in deeper tissue and a more targeted effect on cancerous areas. This significantly increased the effectiveness of PDT. However, the greatest progress has been made in the case of third-generation photosensitizers. By combining photosensitizers with biomolecules or encapsulating them in carriers, it has become possible to significantly increase the selectivity of PDT towards cancer cells. These photosensitizers can target specific molecules or structures in cancer cells to overcome side effects [167,168,169,170]. In particular, 5-ALA has gained recognition as a beneficial photosensitizer in gliomas [171,172,173,174,175]. As a porphyrin precursor, 5-ALA is metabolized through the heme biosynthetic pathway to produce protoporphyrin IX (PpIX), a tumor-selective photosensitizer [98,176,177]. The high tumor selectivity for fluorescent protoporphyrin IX (PpIX) accumulation after systemic administration of 5-ALA enables intraoperative fluorescence guidance [178,179,180,181]. The advantages of 5-ALA over other photosensitizers are compelling. Numerous studies highlight the effectiveness of 5-ALA-based PDT in the treatment of gliomas, achieving cytotoxicity levels up to 80% in vitro and high tumor specificity [172,173,182,183,184,185]. PDT using 5-ALA has been shown to be safe at doses of 90 mg/kg or less followed by irradiation of rat brains with light intensity of 100 J/cm^2^ [186].

## 5. Application

### 5.1. In Vivo Photodynamic Therapy

#### 5.1.1. In Human

Perria and colleagues were the first to use PDT for glioma. Many neurosurgeons began treating glioma patients with PDT [187,188,189,190,191,192,193]. Fluorescence-guided tumor resection followed by PDT has been observed to prolong mean survival in patients diagnosed with glioma [194,195,196,197]. One of the first randomized, controlled trials of PDT in the treatment of malignant gliomas, conducted by Muller and Willson, included 43 glioma resections followed by PDT with photophrin, as well as a control group of 34 patients who underwent tumor resection without PDT. Median survival was 11 months in the treated group and 8 months in the control group. An increase in median survival after PDT by 38% and a higher 6-month survival rate in the treated group were demonstrated [31,196,198]. In the phase II study conducted by Kostron et al., after obtaining informed consent, 26 patients diagnosed with recurrent WHO grade IV glioma were treated with PDT using the PS Foscan (biolitec Pharma, Jena, Germany). Before treatment, all cancers were progressing and standard therapeutic options (irradiation, chemotherapy) had been exhausted. After aggressive fluorescence-guided resection (macroscopically completed in 75% of cases), intraoperative PDT was used. The median time to progression after surgery was 6 months, the median survival was 8.5 months, and the 2-year survival rate was 15%. Compared to matched controls, significantly improved survival outcomes were demonstrated in the treated group [196,199].

One study assessed the effect of photodynamic therapy and a monoclonal antibody against the vascular endothelial adhesion molecule on glioma growth in vivo. Seven days after implantation of glioma cells, adult male rats with glioma were randomly assigned to study groups as follows: (1st group) PDT + anti-vascular endothelial adhesion molecule monoclonal antibody, (2nd group) PDT, (3rd group) anti-vascular endothelial adhesion molecule monoclonal antibody, and (4th group) control. Eight days after implantation, the photosensitizer, hematoporphyrin monomethyl ether was administered, followed by PDT. In the following days, from day 8 to day 16, anti-vascular endothelial adhesion molecule monoclonal antibody was administered intravenously every other day. After 21 days, five rats were selected to be sacrificed and examined. The survival and tumor volume of the remaining 10 rats in each group were recorded. In the 3rd group after PDT, inhibition of tumor growth (67.2%) was noticed and prolongation of survival (89.3%) was observed. These effects were even more pronounced in the PDT + monoclonal antibody against vascular endothelial adhesion molecule group. It was found that PDT in combination with a monoclonal antibody against the vascular endothelial adhesion molecule effectively inhibits glioma growth and prolongs survival [200].

In 2013, Muragaki and colleagues conducted a study to evaluate the effectiveness of PDT using sodium talaporfin with the use of a 664-nm semiconductor laser. These studies were conducted in patients with primary malignant parenchymal brain tumors. Twenty-seven people were included with recurrent glioma and received a single dose of PDT with talaporfin sodium before surgery. After surgery, the area after resection were irradiated with a semiconductor laser with a wavelength of 664 nm between 22 and 27 h after PS delivery. This study was performed on 22 patients. A 12-month survival (more than 95%), 6-month progression-free survival (91%), and 6-month local progression-free survival (91%) were noticed. Incorporating intraoperative PDT into a combined treatment strategy may have a positive impact on average survival, especially in patients with newly diagnosed glioma [201].

A randomized, controlled phase III trial by the Eljamel Group evaluated fluorescence-guided resection using 5-ALA and Photofrin in repetitive PDT where 27 patients were recruited. There were 13 people in the study group with an average survival of 52.8 weeks and 14 people in the control group with an average survival of 24.6 weeks. There were no differences in complications and hospital stays between these groups. The average time to cancer progression was 8.6 months in the study group compared to 4.8 months in the control group. Therefore, it was shown that 5-ALA and Photofrin fluorescence-guided resection and repeated PDT provided a significant improvement in survival without additional risk to glioma patients [202]. A study by V. Turubanova and her team represents a significant step in understanding the potential of PDT in the context of murine glioma and fibrosarcoma. They used PSs such as a mixture of di-, tri-, and tetrasubstituted fractions of aluminum phthalocyanine and photodithazine (bis-N-methylglucamine salt of chlorin e6) in the induction of cancer cell death in response to light irradiation with a wavelength of 615 to 635 nm at a light dose of 20 J/cm^2^.

In Parkinson’s disease, PS accumulates mainly in the endoplasmic reticulum and the Golgi apparatus. Parkinson’s disease PDT-induced cell death was inhibited only by zVAD-fmk. The most interesting aspect of this study is that dying tumor cells induced by PDT using both PS and PD emitted specific molecular signals such as calreticulin, HMGB1, and ATP. Moreover, these cells were bone marrow-derived dendritic cells (BMDCs). Importantly, dendritic cells matured, became activated, and began to secrete interleukin 6 (IL-6) [203].

A study by Sheng-Li Hu and colleagues contributed to research on the therapeutic potential of PDT in the context of the treatment of C6 glioma, which is important due to its aggressive nature in the context of brain tumors. The aim of this study was to thoroughly understand the effect of PDT on the homeostasis of calcium (Ca^2^⁺) and potassium (K⁺) ions in C6 glioma cells and what mechanisms influence the survival of these cells.

This study identified five experimental groups of C6 glioma cells:

1. A control group that did not undergo any form of treatment or radiotherapy.
2. A group in which cells were treated with a hematoporphyrin derivative (HpD) at a concentration of 10 mg/L but not irradiated with light.
3. PDT group in which cells were treated with HpD (10 mg/L) and irradiated with light.
4. PDT&CNQX group, in which cells were treated with HpD (10 mg/L) and the AMPA glutamate receptor antagonist, i.e., CNQX (at a concentration of 50 mol/L), and then subjected to PDT.
5. HpD&CNQX group in which cells were treated with HpD (10 mg/L) and CNQX (50 mol/L) but not irradiated with light.

The research results clearly showed that PDT causes a significant inflow of calcium ions (Ca^2^⁺) into C6 glioma cells and an outflow of potassium ions (K⁺), which ultimately leads to the death of these cells. An interesting finding was that treatment with an AMPA receptor antagonist (CNQX) before PDT partially blocked these changes in ion homeostasis while increasing cell survival. These observations suggest that disturbances in the homeostasis of calcium and potassium ions play a key role in the mechanisms of action of PDT on C6 glioma cells. This discovery is important for further understanding the complex mechanisms of action of PDT and its role as a potential therapeutic method in the treatment of glioma and other brain tumors [204]. The study by Deng-Pan Wu and his team focused on the therapeutic potential of PDT in the treatment of glioma. Intercellular communication (GJIC) was studied, in which the Connexin (Cx)43 protein plays a key role. Intercellular communication plays an important role in transmitting signals leading to apoptosis, i.e., programmed cell death, which may increase the effectiveness of therapies, including chemotherapy and gene therapy. The study results showed that Cx43-mediated GJIC significantly increases PDT phototoxicity, both in laboratory conditions (in vitro) and in living organisms (in vivo). This finding is significant and suggests the potential of using GJIC to improve the effectiveness of PDT. Of particular interest is that GJIC with Cx43 significantly increases PDT phototoxicity in U87 glioma cells that express Cx43. Blocking Cx43 expression had a negative impact on this effect. Additionally, the presence of GJIC with Cx43 contributed to a reduction in tumor diameter and mass after PDT using the PS Photofrin. The effectiveness of PDT with GJIC with Cx43 has been linked to the activity of stress signaling pathways such as the generation of ROS, calcium, and lipid peroxide. These observations suggest that the presence of GJIC, especially involving Cx43, may significantly increase PDT phototoxicity, which is important in the context of glioma treatment. This discovery opens prospects for further development of therapeutic strategies that aim to increase Cx43 expression or enhance GJIC function with Cx43. This, in turn, may lead to an increase in the sensitivity of cancer cells to PDT and improve its effectiveness as a therapeutic method. It is also worth noting that blocking Cx43 expression or GJIC function with Cx43 may influence the development of tumor cell resistance to PDT [205]. The aim of the study by R. Kammerer and colleagues was to investigate changes in the transcriptome of human glioma cells (U87, U373) after non-lethal dynamic phototherapy (PDT) both in vitro and in vivo. The results of the study showed that after PDT, the most increased gene expression encoded proteins related to pathways activated by cellular stress and proteins involved in cell cycle arrest. This response resembled that of cancer cells to high-dose PDT. As a result, PDT affected glioma cells by activating stress response pathways, which led to the inhibition of their cell cycle. However, there were other important observations. Cancer cells after immortalized PDT significantly upregulated a number of immune-related genes, including chemokine genes (CXCL2, CXCL3, IL8/CXCL8) as well as genes encoding interleukin-6 (IL-6) and its receptor (IL6R). These genes can stimulate inflammatory responses. Of note, IL-6 and IL6R may also influence tumor growth. Therefore, these results suggest that PDT may support the immune response against the tumor even if it is unable to completely eliminate it through phototoxic mechanisms alone. However, there is also the potential to stimulate autocrine loops that promote tumor growth, as seen in increased expression of IL-6 and its receptor. His finding suggests that despite the beneficial effects of PDT on the immune system, there may also be a risk of stimulation of tumor growth by nonlethal PDT.

Therefore, the study highlights the complexity of PDT interactions with tumor cells and the need for further research on optimal therapeutic strategies and control of inflammatory responses in the context of PDT treatment of glioma [206]. The study by Fisher and colleagues represents an important step in PDT research in the context of treating glioma, an aggressive brain tumor. The aim of the study was to determine whether mild hypothermia could influence the effectiveness of PDT by increasing the killing of glioma tumor cells while protecting normal neuronal structures. The study consisted of in vitro studies conducted on neuronal cells and in vivo studies conducted on rat models. The results of in vitro studies showed that hypothermia significantly increases the survival of neuronal cells after the use of PDT. In vivo studies in rats confirmed that hypothermia has a protective effect on neural structures after PDT, as reflected in T_2_ mapping results that showed a reduction in the volume of edema and inflammation in the brain. One of the key results was an increase in protoporphyrin IX (PpIX) fluorescence in brain tumors after hypothermia. This, in turn, had a beneficial effect on the survival of rats after PDT. Histological and immunohistochemical analysis showed that hypothermia was an effective method of protecting normal brain structures during PDT. The conclusions from this study suggest that mild hypothermia may significantly improve the effectiveness of photodynamic therapy in the treatment of glioma. Hypothermia may protect neural tissues and simultaneously increase the phototoxicity of PDT, leading to the longer survival of rats after treatment. This discovery is of great importance and opens the prospects for further research on the use of hypothermia as an effective therapeutic strategy in the treatment of glioma [207]. Zhang’s study used cell membranes to encapsulate indocyanine green (ICG) nanoparticles (SLNP/ICG), referred to as SLNP/ICG@M, for targeted glioma PDT. Moreover, SLNP/ICG@M produce a large amount of ROS under NIR irradiation. SLNP/ICG@M with NIR irradiation can activate the mitochondrial-mediated apoptosis pathway [208]. It is known that high concentrations of cellular glutathione (GSH) in cancer cells can reduce the ability of PDT to selectively destroy the tumor, so it is necessary to find a way to improve the therapeutic ratio of PDT in brain tumors. In a study by F Jiang et al., PDT using Photofrin as a photosensitizer combined with administration of buthionine sulfoximine (BSO), an agent that depletes glutathione levels in BSO cells, was performed in male intracerebral U87 and healthy Fisher rats. In tumor-bearing U87 rats, in vivo treatment with the PDT-BSO combination showed significantly greater tumor necrosis than individual treatment [209]. In the study by Yi et al., 24 rats with subcutaneously implanted C6 rat glioma of similar size were randomly divided into 3 groups: group 1—receiving 5-ALA-PDT, group 2—laser irradiation, and group 3—sham procedures but no treatment. Compared with groups 2 and 3, the volume of tumor grafts was significantly reduced (*p* < 0.05), MVD was significantly reduced (*p* < 0.001), and cell necrosis was significantly increased in group 1. The underlying mechanism may involve increasing cell necrosis but not inducing cellular apoptosis, which may result from the destruction of tumor micro-vessels [210]. Clinical trials have shown that PDT significantly increases the median survival of patients with gliomas. Experimental studies have shown that increasing the optical energy and PS dose leads to an increase in the volume of tumor necrosis. However, increasing the dose of light delivered to the tumor may increase the risk of causing permanent neurological deficits. In the study by Zheng et al., the neuroregenerative effect of atorvastatin on PDT was examined. However, atorvastatin significantly reduced PDT-induced cell damage. To further investigate the mechanisms underlying atorvastatin-mediated reduction in functional deficits, the effects of atorvastatin on angiogenesis and synaptogenesis were examined. Atorvastatin was found to significantly induce angiogenesis and synaptogenesis in PDT-damaged brain tissue [211]. The effectiveness of PDT is known to be induced by ROS production, dependent on hypoxia. Moreover, hypoxia activates sodium hydrogen exchanger 1 (NHE1), which is an essential component of tumor progression and metastasis. The study by Hou et al. showed that PDT significantly inhibited primary tumor growth. These results suggest that PDT with DHA may increase total ROS levels to attenuate glioma invasion and migration by suppressing NHE1 expression. The study by Kuiyuan Hou et al. showed that PDT significantly inhibited primary tumor growth, whereas PDT in synergy with DHA also inhibited recurrent tumors and improved overall survival by regulating the ROS-NHE1 axis. No visible side effects were observed. These results suggest that PDT with DHA may increase total ROS levels to attenuate GC invasion and migration by suppressing NHE1 expression [212].

In the study by Terzis et al., the effect of PDT on glioma cells (GaMg and U-251 Mg) was checked. Directional cell migration and spheroid growth were determined for both cell lines exposed to increasing laser output power (15–35 J/cm^2^) at Photosan-3 photosensitizer concentrations of 5 and 7 micrograms/mL. This effect occurred within the first 4 days after exposure to the drug. Spheroids from both cell lines also showed drug dose- and laser-power-dependent growth inhibition that became apparent after a 6-day lag period. During this period, the outer layers of the spheroid cells disintegrated. The remaining spheroid tissue was unable to migrate and regrow under the highest laser energy (30–35 J/cm^2^, 5 and 7 micrograms/mL Photosan-3). However, these spheroids have demonstrated the ability to invade when confronted with normal brain cells aggregated in vivo [213]. Table 1 presents examples of clinical studies with some PSs in glioma.

#### 5.1.2. In Animal Model

The study by Han-Wen Guo and colleagues used a low fluence rate and implemented long-term photodynamic therapy (PDT) in a mouse model of human glioma using an organic light-emitting diode. A single dose of 5-aminolevulinic acid (ALA) was used as a photosensitizer. Tumor volume was monitored by bioluminescence imaging and animal survival time was recorded. It was found that in animals with comparable tumor volume before and immediately after irradiation, the mean overall survival of mice subjected to PDT was 40.5 ± 9.2 days, which significantly exceeded the survival time of control mice (26.0 ± 2.0 days) [227].

Another study assessed the effects of photodynamic therapy on glioma and brain tissue. Following clinically relevant doses of PDT, reductions in tumor volume, glioma cell proliferative activity, and vascular endothelial growth factor expression were observed in the tumor area and adjacent brain tissue for 7 days. Twenty athymic mice underwent implantation of glioma cells. Fifteen mice were administered Photofrin intraperitoneally on day 6 after tumor implantation and then subjected to laser therapy with different optical doses (40 J/cm^2^, 80 J/cm^2^, and 120 J/cm^2^) after 24 h post Photofrin injection. The remaining five tumor-bearing mice served as controls. All animals were sacrificed 14 days after tumor implantation. It was found that the tumor volume in the group of mice receiving 80 or 120 J/cm^2^ was significantly smaller than in the control group. Vascular endothelial growth factor immunoreactivity in adjacent brain tissue increased significantly in mice treated with 120 J/cm^2^ PDT compared to the control group, as well as in mice treated with Photofrin and lower optical doses. No significant differences in glioma cell proliferation and vascular endothelial growth factor expression in the tumor area were observed between groups. These results suggest that PDT is effective in tumor shrinkage, especially with higher light doses, and PDT-induced vascular endothelial growth factor expression in adjacent brain tissue may be associated with tumor recurrence. Therefore, combining PDT with antiangiogenic drugs may be an effective strategy for the treatment of glioma [228]. In order to evaluate the effectiveness of the synthesized nanoparticles dedicated to PDT glioma, athymic mice were bred. Glioma was induced by subcutaneous injection of U87-MG cells. PDT was performed 15 days after implantation. CPN was administered intravenously and intratumorally. Mice treated with a single i.t. or intravenous CPN administration showed strong tumor growth inhibition for 10 days after PDT treatment. Tumors from the PBS control or nonirradiated CPN groups reached more than twice their initial volume [229].

Human monocyte cells (THP-1) and mouse monocytes isolated from bone marrow (mBMDM) were used as covert CPN carriers to penetrate glioma spheroids and an orthotopic tumor model. The success of PDT using this cell-mediated targeting strategy was determined by its effect on spheroids. CPNs did not affect monocyte viability in the absence of light and did not show nonspecific release after cell loading [230].

## 6. PDT Glioma Current Clinical Research

Photodynamic therapy is approved by the US Food and Drug Administration (FDA) for the treatment of premalignant diseases and malignancies such as actinic keratosis, Barrett’s esophagus, esophageal cancer, and non-small cell lung cancer [171]. Many of the novel concepts and strategies for using PDT in the treatment of glioma are in the in vitro experimental phase, still requiring extensive evidence of effectiveness studies before clinical application [9]. A study by Vermandel et al. examined the safety and efficacy after intraoperative treatment of glioma with photodynamic therapy (PDT) after administration of 5-ALA acid and maximal resection in 10 patients with newly diagnosed glioma. There were no serious adverse events, and it was shown that this strategy could help reduce the risk of recurrence by targeting residual tumor cells in the resection cavity, as the 12-month recurrence-free survival rate was 60% [231]. An ongoing study in Germany is evaluating stereotactic biopsy followed by 5-ALA-based stereotactic PDT and the feasibility of 5-ALA in stereotactic interstitial PDT in a subset of adult glioma patients [9]. The only fluorescence-guided glioma surgery agent approved by the FDA [232] is 5-ALA.

**Figure 3:**
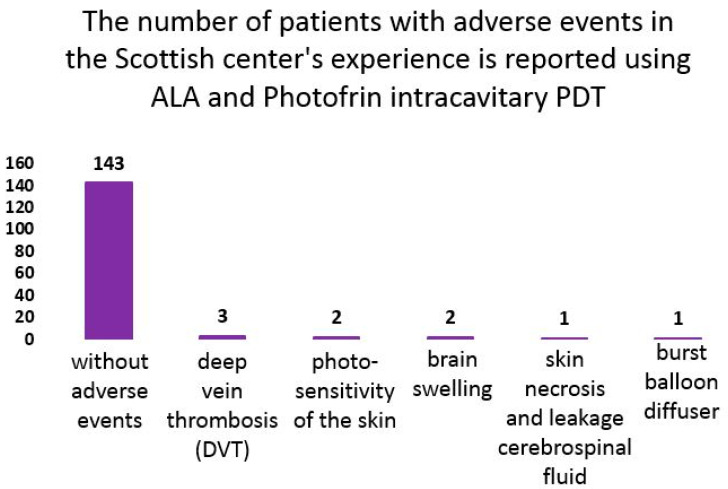
Adverse events in the Scottish center study using ALA and Photofrin intracavitary PDT with a balloon diffuser and a 630 nm diode laser on brain tissue.

In Scotland, over 365 brain treatments were performed in patients using 5-ALA and Photofrin intracavitary PDT with a balloon diffuser and a diode laser with a wavelength of 630 nm. Of these treatment cases, 143 had no adverse effects (Figure 3). Adverse events were reported in seven patients: three patients developed deep vein thrombosis (DVT), two patients experienced skin photosensitivity due to lack of light protection, two patients experienced cerebral edema after PDT, one patient experienced skin necrosis and cerebrospinal fluid leakage, and in one patient the balloon diffuser ruptured due to poor catheter attachment [124,170].

These side effects are no different from those occurring during other therapies [233,234]. Research conducted on 20 patients treated with HPD and 630 nm light revealed five expected skin photosensitivities, none of which were serious adverse events [233,234,235]. Only 2 of 136 patients treated with HPD-PDT in Australia (2%) reported excessive sunburn associated with skin photosensitivity [236]. In both cases, they did not follow the recommended instructions. The main side effect associated with treatment is short-term hypersensitivity of the skin and retina to light after the administration of a PS [196].

## 7. Conclusions

Research on PDT in the treatment of gliomas is a fascinating area of medical research that opens new perspectives in the fight against this aggressive form of brain cancer. Previous and current in vivo studies and patient clinical experiences show promising results with PDT as an effective and increasingly popular therapeutic option in the treatment of gliomas. The most important finding from this study is the significant improvement in median survival for patients who received PDT as part of their treatment. Prolonged progression-free survival has been observed in many cases, which is an important step in the fight against this serious disease. These results suggest that PDT may provide a valuable alternative to traditional glioma treatments such as surgery, radiotherapy, and chemotherapy, which are often associated with significant side effects and limited effectiveness. Additionally, research on PDT has shown that this therapy can stimulate the body’s immune response against cancer cells. This finding is particularly promising because it highlights the potential of PDT as a tool to induce a defense response against gliomas, which may further aid in tumor elimination. It is worth emphasizing that the results of in vivo studies on animal models and clinical studies on patients unanimously suggest the benefits of using PDT in the treatment of gliomas. This is important evidence of the promising nature of this therapy and its potential to change the standards of treatment for this difficult disease. Although the results of these studies are encouraging, further research is necessary to optimize PDT parameters, including the selection of appropriate photosensitizers, light parameters, and combination treatment strategies. Moreover, a key challenge is to adapt PDT to the individual patient’s case, taking into account differences in disease stage and molecular caesuras. These conclusions emphasize the importance of continuing research on PDT as a promising therapeutic strategy for patients suffering from gliomas. Further development of this field of medicine may bring significant benefits to patients in the form of improved quality and length of survival, as well as influence future standards of treatment of this difficult and fatal disease.

## Figures and Tables

**Table 1:** Characteristics of selected clinical studies (with PS applied, excitation wavelength and treatment window).

Article	Photosensitizer	Excitation Wavelength (nm)	Treatment Window
[214]	Porfimer Sodium	630	48–150 h
[215]			
[216]			
[217]			
[218]			
[219]	Hematoporphyrin derivative [HpD]	408, 510, 630	24–48 h
[218]			
[220]	Dihematoporphyrin ether [DHE]	395, 630	24–72 h
[221]	5-Aminolevulinic Acid	410, 510, 635	4–8 h
[222]			
[218]			
[218]	Talaporfin sodium	664	12–26 h
[223]	Temoporfin [m-THPC; m-tetrahydroxyphenylchlorin]	652	48–110 h
[218]			
[224]	Boronated protoporphyrin [BOPP]	630	24 h
[218]			
[214]	Benzoporphyrin derivative [BPD]	680–690	15–30 min
[225]			
[226]			
